# Effects of Long-Term CO_2_ Enrichment on Soil-Atmosphere CH_4_ Fluxes and the Spatial Micro-Distribution of Methanotrophic Bacteria

**DOI:** 10.1371/journal.pone.0131665

**Published:** 2015-07-06

**Authors:** Saeed Karbin, Cécile Guillet, Claudia I. Kammann, Pascal A. Niklaus

**Affiliations:** 1 Institute of Evolutionary Biology and Environmental Studies, University of Zürich, Zürich, Switzerland; 2 Institute of Plant Ecology, Justus-Liebig-University, Giessen, Germany; 3 Climate Change Research for Special Crops, Hochschule Geisenheim University, Geisenheim, Germany; University of Copenhagen, DENMARK

## Abstract

**Background:**

Effects of elevated atmospheric CO_2_ concentrations on plant growth and associated C cycling have intensively been studied, but less is known about effects on the fluxes of radiatively active trace gases other than CO_2_. Net soil-atmosphere CH_4_ fluxes are determined by the balance of soil microbially-driven methane (CH_4_) oxidation and methanogenesis, and both might change under elevated CO_2_.

**Methods and Results:**

Here, we studied CH_4_ dynamics in a permanent grassland exposed to elevated CO_2_ for 14 years. Soil-atmosphere fluxes of CH_4_ were measured using large static chambers, over a period of four years. The ecosystem was a net sink for atmospheric CH_4_ for most of the time except summer to fall when net CH_4_ emissions occurred. We did not detect any elevated CO_2_ effects on CH_4_ fluxes, but emissions were difficult to quantify due to their discontinuous nature, most likely because of ebullition from the saturated zone. Potential methanotrophic activity, determined by incubation of fresh sieved soil under standardized conditions, also did not reveal any effect of the CO_2_ treatment. Finally, we determined the spatial micro-distribution of methanotrophic activity at less than 5× atmospheric (10 ppm) and elevated (10000 ppm) CH_4_ concentrations, using a novel auto-radiographic technique. These analyses indicated that domains of net CH_4_ assimilation were distributed throughout the analyzed top 15 cm of soils, with no dependence on CH_4_ concentration or CO_2_ treatment.

**Conclusions:**

Our investigations suggest that elevated CO_2_ exerts no or only minor effects on CH_4_ fluxes in the type of ecosystem we studied, at least as long as soil moisture differences are small or absent as was the case here. The autoradiographic analyses further indicate that the spatial niche of CH_4_ oxidation does not shift in response to CO_2_ enrichment or CH_4_ concentration, and that the same type of methanotrophs may oxidize CH_4_ from atmospheric and soil-internal sources.

## Introduction

The atmospheric concentrations of greenhouse gases including carbon dioxide (CO_2_) and methane (CH_4_) have increased since pre-industrial times due to anthropogenic activities. A question of particular concern is how elevated atmospheric CO_2_ concentrations affect terrestrial ecosystems and their functioning. Studies of plant growth responses and of effects on the carbon balance of ecosystems have dominated elevated CO_2_ research to date. However, although CO_2_-effects are solely mediated by the plant’s photosynthetic apparatus, elevated CO_2_ can influence virtually every plant or microbial process through alterations of the ecosystem’s carbon, nitrogen or water dynamics. An intriguing question is whether these effects will affect the ecosystem’s balance of trace gases other than CO_2_ such as CH_4_. Such a mechanism would interact with global climatic change, similar to effects on carbon sequestration.

The CH_4_ balance of an ecosystem is determined by the sum of sources and sinks, both of which are almost exclusively driven by soil microbial processes [1] (but see [2, 3]). Whether sources or sinks dominate is often determined by oxygen availability, with CH_4_ oxidizing micro-organisms driving soil CH_4_ uptake under aerobic conditions whereas methanogenesis by archaea dominates under anaerobic conditions, e.g. in waterlogged soils. Methanogenesis and CH_4_ oxidation often co-occur, with a substantial fraction of the CH_4_ produced in anoxic soil domains being consumed by methanotrophs before it diffuses to the atmosphere. Under these conditions, methanotrophs functionally act as a “biofilter” for endogenous CH_4_. Conversely, methanogenesis can prime the activity of methanotrophs [4], which then in turn will oxidize larger amounts of atmospheric CH_4_ once the soil-internal sources cease [5]. Oxidation of atmospheric CH_4_ (low concentrations) or soil-internal CH_4_ (high concentrations) requires enzymes with vastly different kinetic properties. Methanotrophic organisms growing at atmospheric CH_4_ concentrations have not been isolated to date, and it therefore remains unclear whether different groups of methanotrophs are responsible for these two sinks or whether the same organisms exhibit different CH_4_ oxidation kinetics by physiological adjustment [5].

The ecology of atmospheric CH_4_ oxidation is not well understood to date. Many studies have shown that gas phase diffusive CH_4_ transport limitations often control soil CH_4_ uptake, at least at moderate to high soil moisture [6]. However, moisture can also limit methanotrophic activity due to physiological stress [7]. A second important factor is nitrogen availability. High mineral nitrogen levels, in particular NH_4_^+^, can inhibit CH_4_ oxidation. Laboratory studies have attributed this effect to inhibition of methane mono-oxygenase, the enzyme catalyzing the first step of CH_4_ assimilation. However, mineral N also is an essential nutrient and the relationship between CH_4_ oxidation and N levels therefore is more complicated [8]. Finally, inhibition of methanotrophic activity does not necessarily translate into reduced soil CH_4_ uptake. [9] have demonstrated that mineral fertilizer N that accumulates under drought (because plant uptake is reduced) can inhibit methanotrophs in the top soil layers, but that methanotrophs in deeper soil layers can compensate for this loss of function (because diffusion is facilitated by low soil moisture), so that no effect manifests in soil surface CH_4_ fluxes.

Elevated CO_2_ concentrations have the potential to affect soil CH_4_ transformations by various mechanisms. First, CO_2_-enrichment is often found to increase soil moisture due to increased photosynthetic water use efficiency [10, 11]. Since soil moisture is an important controller of CH_4_ diffusion rates, CH_4_ oxidation could be reduced by this mechanism. Second, elevated CO_2_ can reduce mineral N availability through increased plant and microbial N uptake and through effects on microbial N transformation rates [12–15], which in turn might alter CH_4_ oxidation. Third, plants exposed to elevated CO_2_ can produce larger amounts of organic compounds that enter the soil via rhizodeposition and litterfall [16]. These could fuel methanogenesis through higher substrate availability and lower redox potential caused by higher respiration rates. Some of these compounds could also directly inhibit methanotrophs, since inhibitory effects have been demonstrated for ethylene [17], some organic acids [18], and terpenes [19].

We studied soil-atmosphere CH_4_ fluxes in a grassland that had been exposed to elevated CO_2_ using free-air CO_2_ enrichment (FACE) for 14 years [20]. Fluxes were assessed with large static chambers. We further determined the spatial micro-distribution of methanotrophs that actively assimilated CH_4_ under low and high CH_4_ concentrations, using a novel auto-radiographic technique. These investigations addressed the following questions: (1) does elevated CO_2_ affect soil-atmosphere CH_4_ fluxes? (2) Does the spatial micro-distribution of active methanotrophs change under elevated CO_2_, and can such effects be related to the observed system-level fluxes? (3) Is the spatial niche of active methanotrophs oxidizing CH_4_ originating from the atmosphere or from soil-internal sources different?

## Methods

### Study site and experimental design

We studied effects of long-term elevated atmospheric CO_2_ on soil-atmosphere CH_4_ fluxes and the micro-distribution of methanotrophic bacteria in a permanent grassland near Giessen, Germany (50°32’ N and 8°41.3’ E, 172 m a.s.l.). For at least the past 50 years, the site has been permanent grassland fertilized with 50–80 kg N ha^-1.^ From 1995 onwards, fertilization was reduced to 40 kg N ha^-1^ a^-1^ (see [20] for further details).

In 1997, three circular plot pairs (FACE rings with 8 m inner diameter) were established. One plot per pair was selected randomly and atmospheric CO_2_ enriched to 20% above ambient conditions during daylight hours since May 1998, using free-air CO_2_ enrichment (FACE). The other plot of the pair served as ambient CO_2_ control.

Vegetation at the site is classified as Arrhenatheretum elatioris Br.-Bl. [21] and contains about 60 vascular plant species [20]. The soil is a Fluvic Gleysol with sandy loam texture over clay. The top soil is slightly acidic (pH of 6.0) and has an organic C content of 4.6% and 3.6% in 0–5 and 5–15 cm depth [20].

### In situ soil-atmosphere CH_4_ fluxes

From 2009 to 2012, we measured soil-atmosphere CH_4_ fluxes on a total of 191 days *in situ* with large static chambers (94 cm inner diameter, ca. 160L volume; modified according to [22]; for further details see [7]). We collected three 25 mL headspace samples at 30 minute intervals and analyzed these by gas chromatography. CH_4_ fluxes were estimated by linear regression of concentrations against time. We accepted all measurements with a residual standard error (RSE) of less than 15 ppb CH_4_, plus the measurements where the ratio of RSE to calculated flux indicated that omission of any of the three points would have changed the result by less than 20%. Measurements that did not fulfil these criteria were analyzed separately, using other methods, as is discussed in the results section.

### Soil moisture and water table depth

Soil moisture was recorded automatically at 4 locations per plot using TDR-probes (P2G, 0–15 cm depth, Imko, Ettlingen, Germany). Water table depth was recorded manually on each weekday, using three custom-built water-level gauges that were placed between pairs of ambient and elevated CO2 plots.

### Soil sampling

On July 6 and October 25, 2011, we harvested two intact soil cores per plot. Cores were sampled with PVC tubes (20 cm depth x 6.5 cm internal diameter) that were driven 15 cm into the soil. In order to minimize soil compaction, the top soil had first been pre-cut along the tube’s circumference with a knife. Cores were then capped at both ends to prevent water loss.

On July 6, 2011, we further collected soil at two random locations per plot. These samples were divided by five centimeter depth interval, down to a depth of 20 cm. The two replicate samples per plot were combined per depth layer and transported to the laboratory for further analysis.

### CH_4_ oxidation of sieved soil samples

We sieved the soil samples (2 mm mesh) and determined soil moisture gravimetrically (5 g fresh soil, 105°C, 24 h). Fresh soil equivalent to 100 g dry weight per plot and depth layer was incubated at 20°C in 1 L air-tight glass jars. The jars were ventilated under ambient conditions, and headspace CH_4_ concentration determined 0, 2, and 4 h after closing of the lids. CH_4_ uptake rates were calculated by linear regression against sampling time.

### Radiolabelling of intact soil cores

The intact soil cores collected at the field site were placed in gas-tight 3 L jars (with the bottom end of the tube still capped). The jars were closed and headspace samples analyzed for CH_4_ after 0, 2, 4 and 6 h to determine the core’s net CH_4_ uptake rates.

The jars were then ventilated and the soil cores labelled with ^14^CH_4_. Two soil cores per plot and sampling date were labelled at slightly above-ambient CH_4_ concentrations (max. 10 ppm). Two additional soil cores from the July 6, 2011 sampling were labelled at high CH_4_ concentrations (ca. 10000 ppm). The rationale of this procedure was to test for differences in spatial activity distribution under these contrasting conditions. A total ^14^C activity of ca. 100kBq was applied over a period of 6 days. Plastic tubes with 100 mL 1M NaOH were placed in each jar to trap CO_2_ (incl. ^14^CO_2_) produced by microbial respiration. We regularly injected O_2_ into the jars to maintain O_2_ concentrations around 20%.

Then, the soil cores were freeze-dried and impregnated with epoxy resin (Laromin C 260, BASF, Ludwigshafen, Germany, mixed at a ratio of 2:3 with Araldite DY 026SP hardener, Astorit AG, Einsiedeln, Switzerland) as described in [9]. The resin was left curing at room temperature for 3 days, followed by an overnight incubation at 60°C for final hardening. The soil cores were then cut twice vertically using a diamond saw, creating a section of ca. 8 mm thickness. This section was cut into three equal pieces which were glued onto 5 × 5 cm glass carriers and levelled with a diamond cup mill (Discoplan, Struers GmbH, Birmensdorf, Switzerland).

We exposed phosphor imaging plates (BAS III S, Fuji Photo Film Ltd., Tokyo, Japan) to levelled soil sections for 3 days. The imaging plates were then digitized by red-excited fluorescence scanning at a resolution of 200 μm (BAS-1000, Fujix corp., Tokyo, Japan). We corrected the scans for background exposure and recombined the three image sections to a single image of the cross-sectional area of the original soil cores, using custom Matlab scripts (Image processing toolbox, Matlab, Mathworks, Natick, MA). The sections were inspected visually, and the vertical distribution of the label calculated by averaging pixel values by horizontal pixel line (excluding large stones).

### Statistical analysis

The unit of replication for the elevated CO_2_ treatment is the field plot. We therefore analyzed the data using one-way ANOVA with CO_2_ treatment as fixed effect and field plot (n = 6) as replicate. We considered pairs of plots (“block” factor) and the geographical northing and easting to account for spatial variation, but these terms consumed excessive degrees of freedom given the small sample size, and did not change the results, so that we did not include them in the final model. Effects with P≤0.05 are referred to as significant, effects with P≤0.1 as marginally significant.

## Results

### In situ soil-atmosphere CH_4_ fluxes

**Fig 1:**
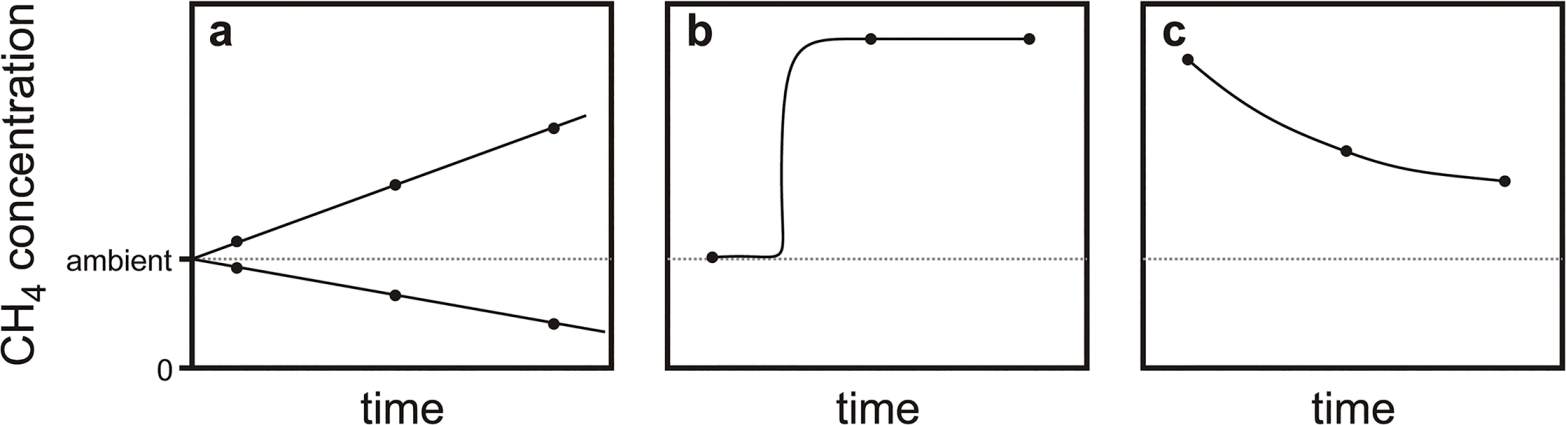
Typical time-courses of CH_4_ concentrations during static chamber sampling. (a) Linear concentration changes with time, indicating continuous soil CH_**4**_ uptake or release. (b) Step-increase in CH_**4**_ concentration, likely caused by emission bursts that could originate from ebullition from the underlying saturated zone. (c) Decrease in CH_**4**_ concentrations, starting at substantially above-ambient CH_**4**_ concentrations; this pattern is likely caused by a re-distribution of localized CH_**4**_ emissions trapped in the static chamber.

Our static chamber measurements (S1 Dataset) revealed three characteristic patterns in which CH_4_ concentrations evolved over the three headspace samplings (Fig 1). During the major part of the measurements, concentrations progressed linearly with time (Fig 1A), either decreasing from ambient to sub-ambient CH_4_ concentrations (net soil CH_4_ uptake), or increasing to a few hundred to thousand ppb above ambient concentrations (net soil CH_4_ emission). However, in other cases, episodic emissions resulted in a sudden increase of concentrations between some of the headspace samplings (Fig 1B, here shown for emission between 1^st^ and 2^nd^ headspace sampling). We refer to these cases as “bubble emission” since they are likely caused by ebullition from deeper soil layers or the water table. Finally, we also observed CH_4_ concentrations that were markedly above ambient at the first sampling and decreased thereafter (Fig 1C). We termed this pattern “redistribution” since it is likely caused by a localized “bubble emission” prior to the first sampling, followed by redistribution of CH_4_ in the chamber and soil pore volume. There were also cases suggesting a combination of “bubble emission” and “redistribution”, but these were more difficult to classify.

**Fig 2:**
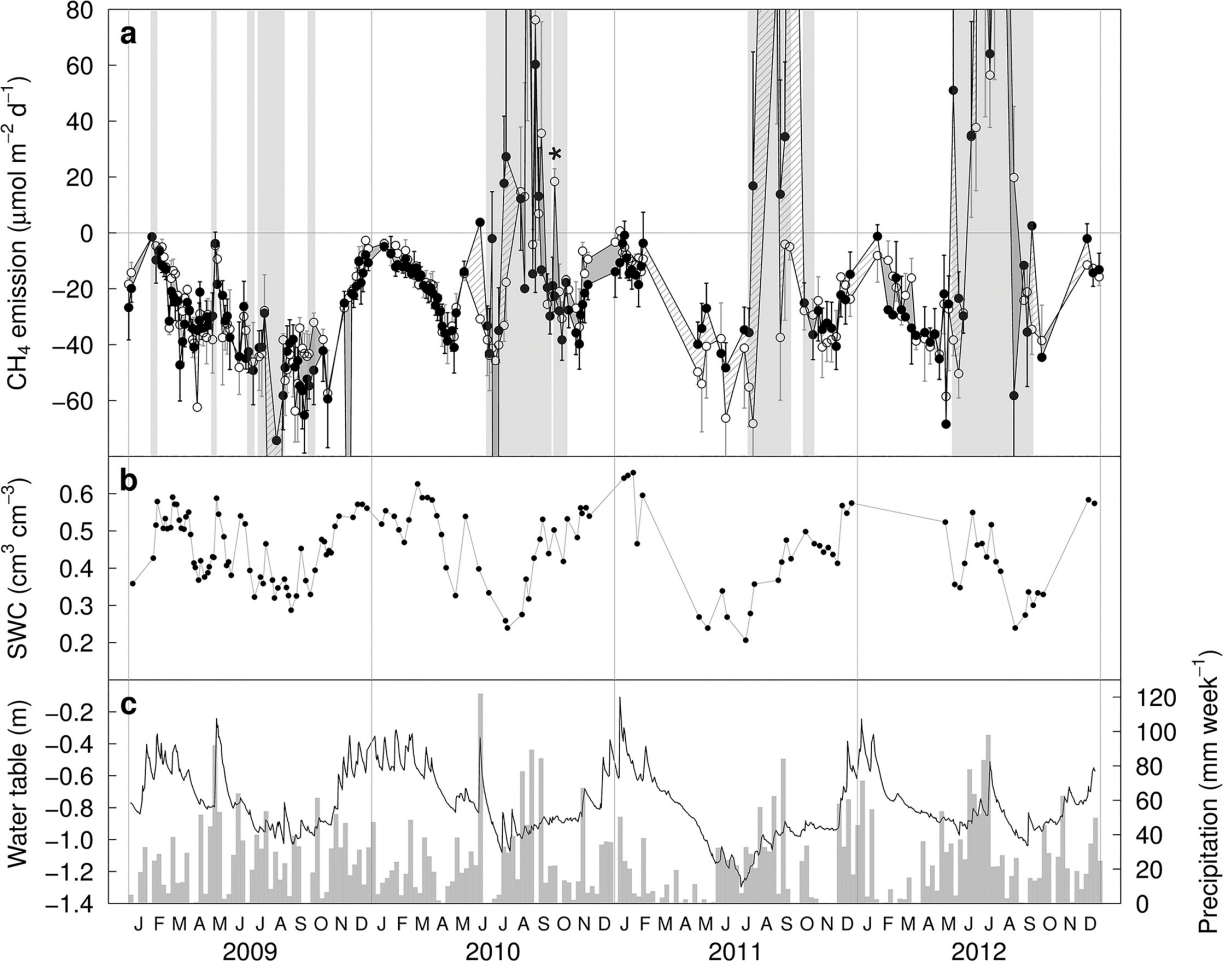
CH_4_ fluxes and related environmental data. (a) CH_**4**_ emission rates in ambient (○) and elevated CO_**2**_ (●) plots, calculated when concentration changes were linear (mean ± s.e., n≤3 per CO_**2**_, depending on the number of plots with emissions following the pattern of Fig 1A). Effects of elevated CO_**2**_ were not statistically significant. Periods during which emissions occurred (Fig 1B and 1C) are shaded in gray, indicating that emission rates likely are underestimates. (b) Volumetric soil moisture, averaged across CO_**2**_ treatments. (c) Weekly precipitation and water table depth.

Meaningful emission rates can only be calculated for the linear case (Fig 1A). In the absence of non-linear emissions, soils were net sinks for CH_4_ (Fig 2A, white background). Soil CH_4_ uptake during these periods did not differ significantly between CO_2_ treatments (26.2±4.7 and 28.6±5.2 μmol CH_4_ m^-2^ d^-1^ in ambient and elevated CO_2_, respectively). During periods in which “bubble emissions” occurred (Fig 2A, grey background), average rates determined from the remaining chambers showing linear emissions were generally positive, i.e. indicated net soil CH_4_ emissions. These emissions likely are lower bounds of the real fluxes because they do not include the supposedly higher emission rates when “bubbles” are formed.

Soil CH_4_ fluxes (excl. periods with “bubble” emissions) were correlated to soil moisture and water table depth, which explained 37% and 57% of the temporal variation in soil-atmosphere CH_4_ exchange (P<0.001, two extreme flux values excluded, sampling day as replicate, Fig 2B and 2C). Soil moisture and water table depth were highly correlated (r = 0.74). CH_4_ fluxes did not significantly depend on daily precipitation.

Bubble emissions occurred in 14.3 (average of 6 plots) out of 168 samplings, with no significant difference between CO_2_ treatments (P = 0.9, generalized linear model with binomial distribution). Virtually identical results were obtained when the number of static chambers per plot showing such emissions (0 to 3 per plot) was considered instead of simply discriminating between occurrence and absence on a plot basis.

### CH_4_ uptake of incubated soil samples

**Fig 3:**
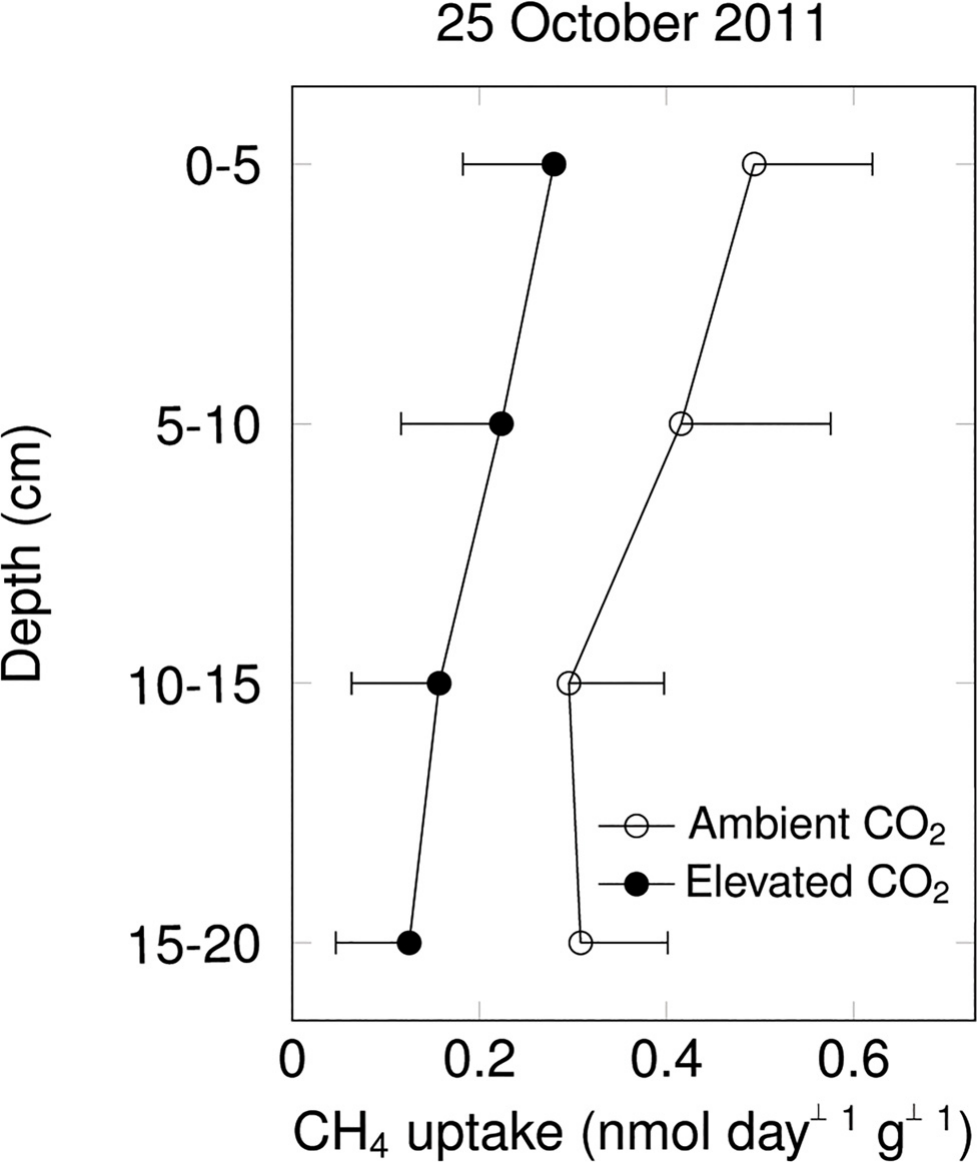
Net CH_4_ uptake rates of sieved field-moist soil incubated at 20°C in the laboratory (mean ± s.e., by 5cm soil layer; n = 3 per CO_2_ treatment; effects of elevated CO_2_ were not statistically significant).

**Fig 4:**
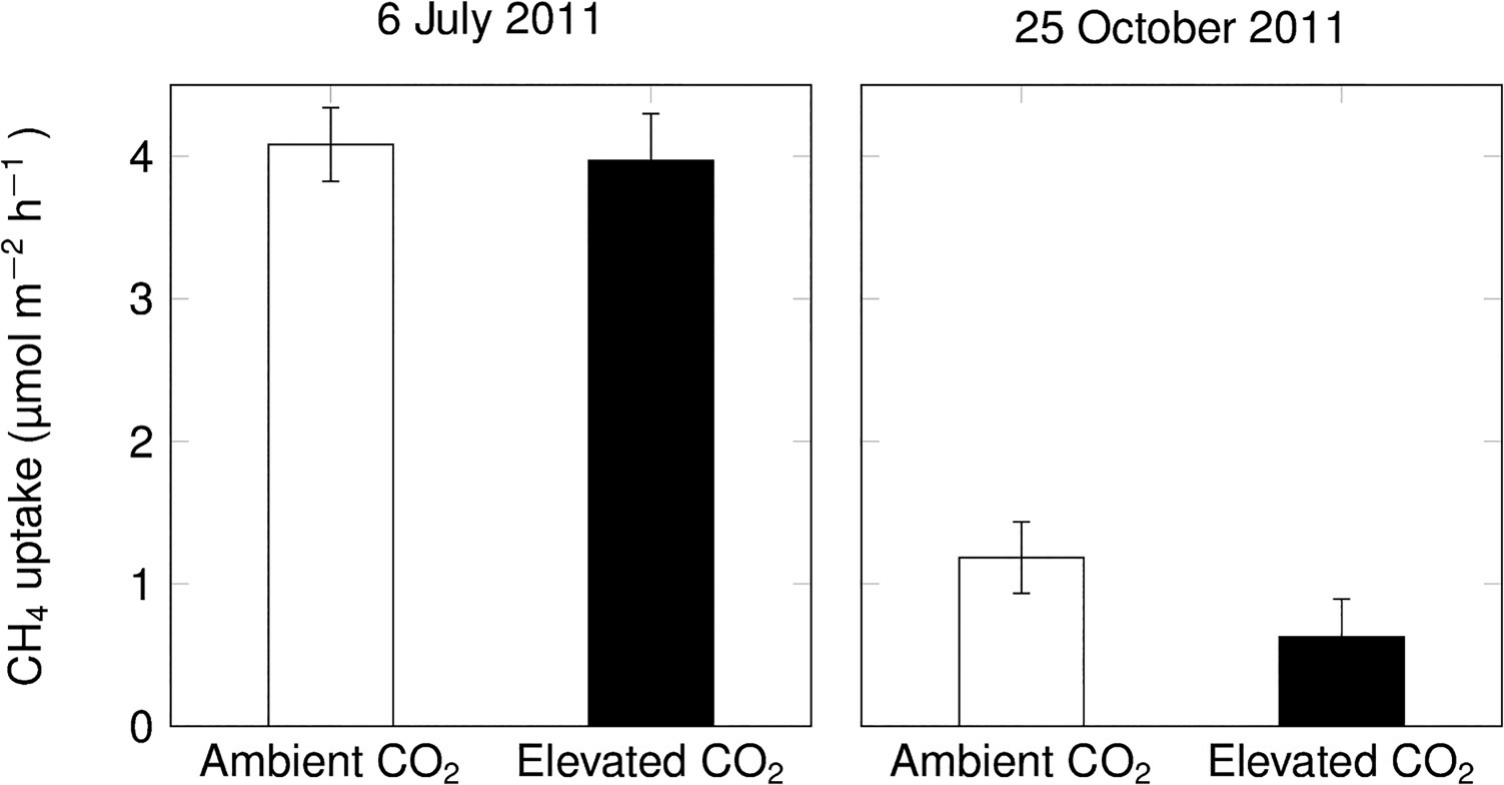
Net CH_4_ uptake rates of intact soil cores collected in ambient and elevated CO_2_ plots and incubated in the laboratory at 20°C (mean ± s.e., n = 3 per CO_2_ treatment; effects of elevated CO_2_ were not statistically significant).

The sieved 5-cm soil layers did not reveal any effect of CO_2_ enrichment when incubated at 20°C and field moisture (Fig 3). Intact soil cores incubated in the laboratory at 20°C also did not show any effect of elevated CO_2_ on net CH_4_ uptake (Fig 4, volumetric soil moisture content of 23% and 46% on July 6 and October 25, respectively).

### ^14^CH_4_ labelling of soil cores

**Fig 5:**
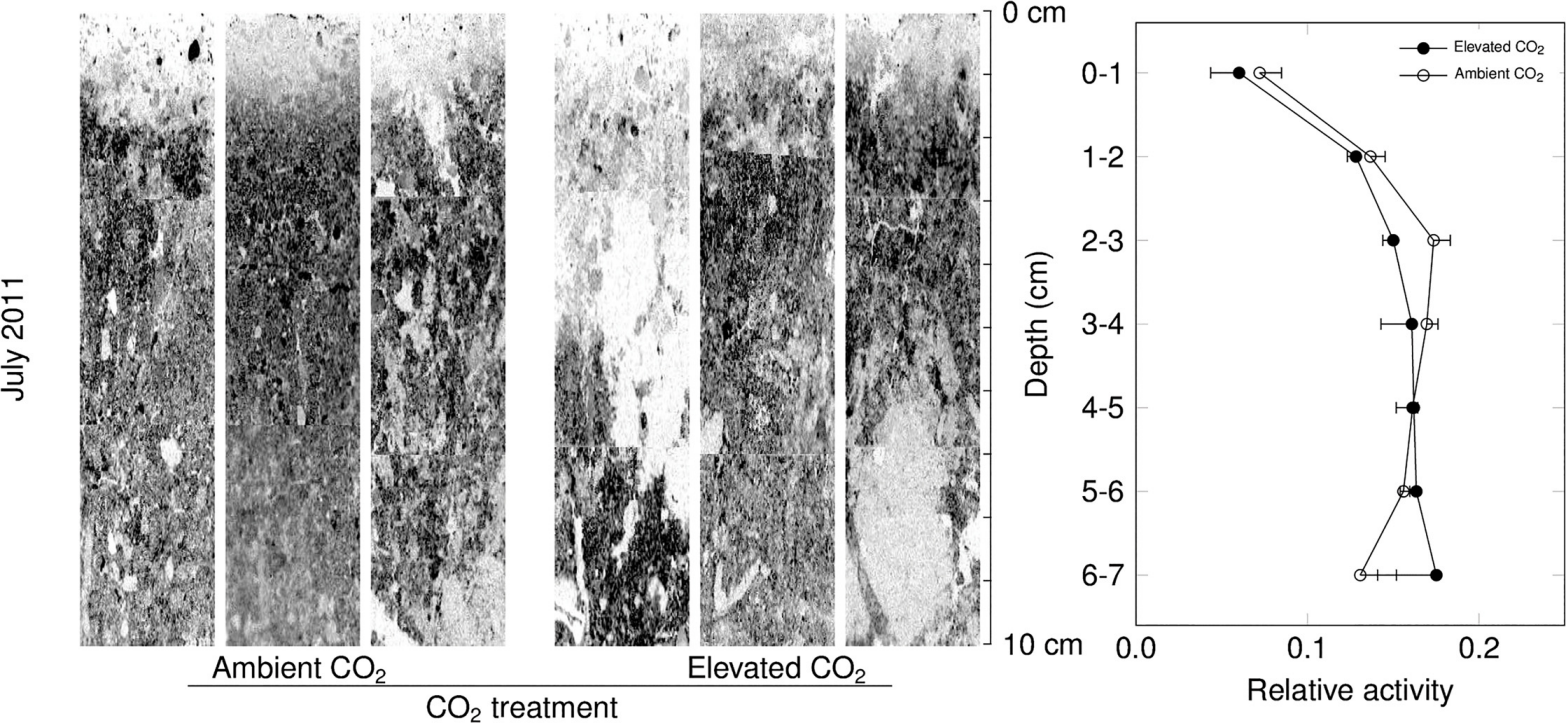
Soil micro-autoradiography of typical soil sections collected on June 6, 2011, and incubated under near-ambient CH_4_ concentrations. Darker pixels indicate higher labelling. Vertical profiles of labelling (right panel), aggregated by 1cm depth intervals (mean ± s.e., n = 3 per CO_**2**_ treatment). Effects of elevated CO_**2**_ were not statistically significant.

**Fig 6:**
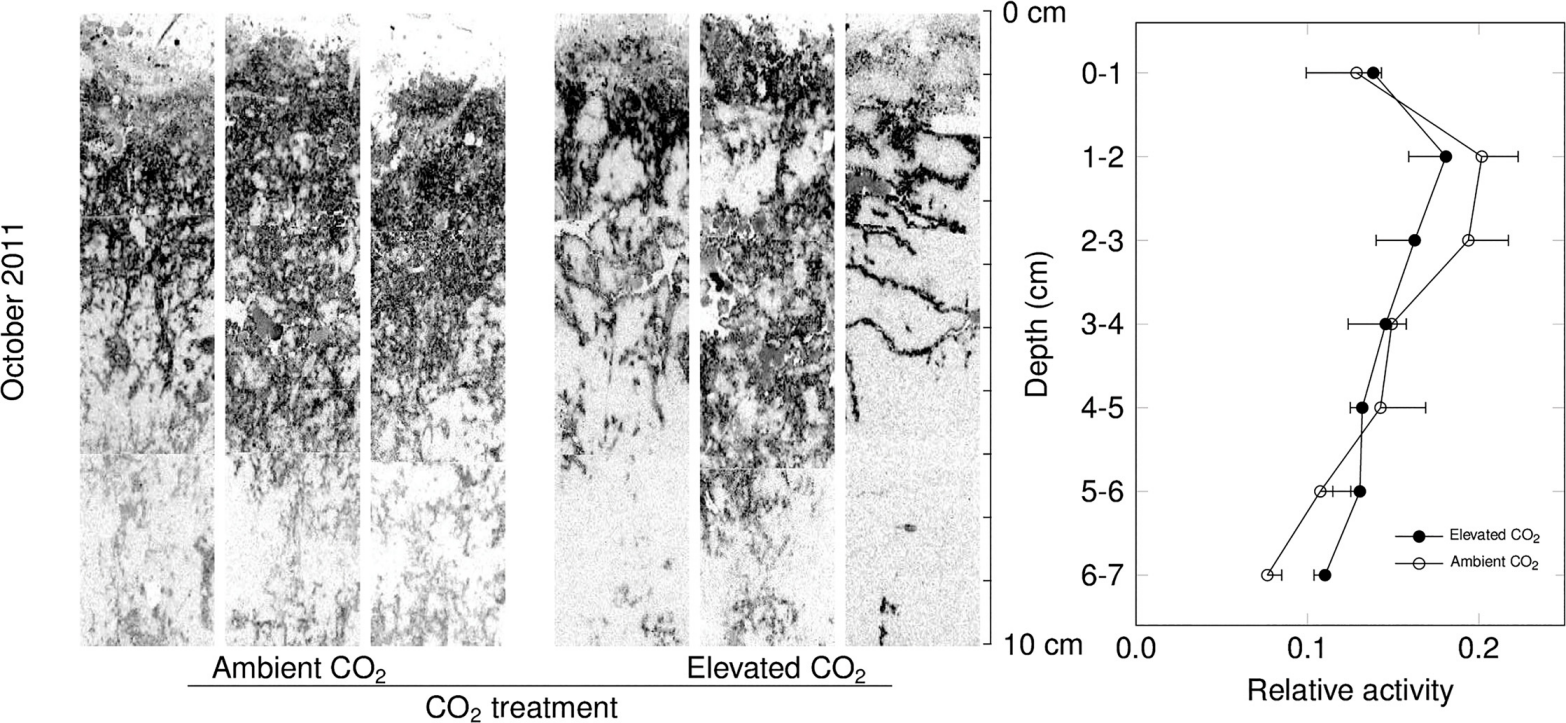
Soil micro-autoradiography of typical soil sections collected on October 25, 2011, and incubated under near-ambient CH_4_ concentrations. Darker pixels indicate higher labelling. Vertical profiles of labelling (right panel), aggregated by 1 cm depth intervals (mean ± s.e., n = 3 per CO_**2**_ treatment). Effects of elevated CO_**2**_ were not statistically significant.

**Fig 7:**
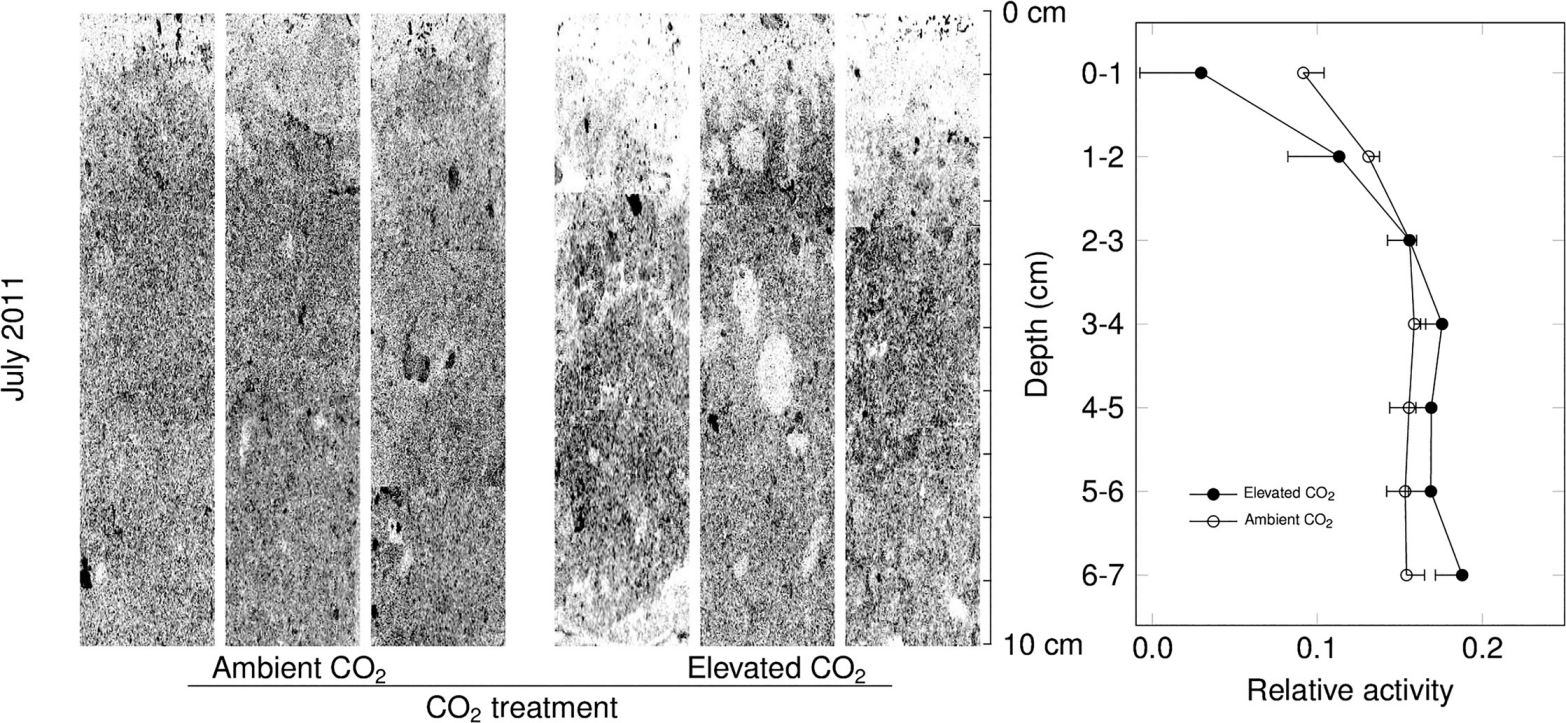
Soil micro-autoradiography of typical soil sections collected on October 25, 2011, and incubated under CH_4_ concentrations around 10000 ppm. Darker pixels indicate higher labelling. Vertical profiles of labelling (right panel), aggregated by 1cm depth intervals (mean ± s.e., n = 3 per CO_**2**_ treatment). Elevated CO_**2**_ marginally significantly affected the depth distribution of methanotrophic activity (P = 0.06 for depth × CO_**2**_).

Visual inspection of autoradiographies revealed heterogeneous label assimilation, with distinct zones of enhanced net CH_4_ assimilation (Figs 5,6 and 7). These appeared to be along cracks and around aggregate structures (e.g. Fig 6). On both July 6 and October 25, net ^14^CH_4_ assimilation was reduced in the top 1–2 centimeters relative to the rest of the soil profile which showed relatively little variation in label intensity with depth.

CO_2_ enrichment did not affect the vertical distribution of the label except for an interaction with depth (P<0.05) that originated from lower labelling of the uppermost layer on October 25 when labelled at high CH_4_ concentration. Since the analysis of depth x CO_2_ treatment includes some degree of autocorrelation of residuals between soil layers, we calculated mean oxidation depth per soil core as

$$\int_{y}\;y∙a\left(d\right)\;dy/\int_{y}\;a\left(d\right)dy,$$

i.e. as activity-weighted mean depth of net CH_4_ assimilation (Table 1); figuratively, this is the depth of the center of gravity an activity depth profile. Mean assimilation depth averaged 3.8 cm, irrespective of CO_2_ treatment and labelling concentration. There was a marginally significant shift of 0.5 cm towards the soil surface in October relative to July 2011 (P = 0.06).

**Table 1:** Oxidation depth (activity-weighted depth of labelling, mean±s.e.) in soil cores from ambient and elevated CO_2_ plots, incubated under low and high CH4 concentrations. Effects of elevated CO_2_ were not statistically significant.

Date	CH_4_ concentration (ppm)	CO_2_ treatment	Oxidation depth (cm)
6 July 2011	10	ambient CO_2_	3.88 ± 0.07
	10	elevated CO_2_	4.00 ± 0.06
25 Oct 2011	10000	ambient CO_2_	3.81 ± 0.08
	10000	elevated CO_2_	4.24 ± 0.42
	10	ambient CO_2_	3.40 ± 0.19
	10	elevated CO_2_	3.45 ± 0.17

## Discussion

In the grassland investigated, soil-atmosphere CH_4_ fluxes were characterized by alternating phases of soil net CH_4_ uptake and emission. On an annual basis, the studied ecosystem was a net source of CH_4_, with emissions peaking during the summer months and oxidation prevailing during most of the remaining time. However, the annual CH_4_ balance is difficult to constrain due to the “burst” character of emissions which is not amenable to the static chamber technique we adopted. We did not detect any effects of elevated CO_2_ on fluxes or micro-distribution of CH_4_ assimilation, but this also may be related to the relatively low power originating from the low replication typical of FACE studies.

Evidence regarding effects of elevated CO_2_ on CH_4_ fluxes is equivocal. In a study in Loblolly pine plantation [23, 24] reductions in soil CH_4_ sink were found under CO_2_ enrichment, which were related to increased soil moisture due to reduced stomatal conductance and increased water use efficiency [25]. The authors argued that this effect on CH_4_ uptake originated from diffusive CH_4_ transport limitation in the top soil but possibly also from increased anoxia in deeper soil layers due to higher plant and heterotrophic soil microbial activity, which could promote methanogenesis. Similar effects were found in trembling aspen stands [26]. Interestingly, in semi-arid grassland, opposite effects of elevated CO_2_ were found when soils were dry [27]; the authors attributed these effects to a reduction of drought stress due to moister soils under elevated CO_2_. This conclusion was supported by soil CH_4_ uptake rates decreasing when soil moisture was above or below some intermediate optimum. However, [28] found reduced CH_4_ uptake under elevated CO_2_ in a mixed *Lolium/Trifolium* sward, and this effect was unrelated to soil moisture. Finally, CH_4_ uptake and CO_2_ concentration were unrelated in a number of other studies (wheat: [29], Sorghum and soybean: [30]; shortgrass steppe: [31]). We observed a median net soil CH_4_ uptake of 23 μmol m^-2^ d^-1^ during periods without emissions. These soil uptake rates are in the upper range of the ones reported in these elevated CO_2_ studies, but not atypical when compared to temperate grassland fluxes reported in an European [32] or global analysis [33]. Elevated CO_2_ did not induce significant changes in soil moisture in our study during the time studied, and it is well possible that CH_4_ fluxes remained unaltered for this reason.

The different character of CH_4_ sources and sinks that contribute to the net balance of the present grassland makes it very difficult to constrain the true annual CH_4_ balance of this ecosystem, for several reasons. First, sink rates due to methanotrophic activity are generally smaller than emissions rates from methanogenesis [34]. Second, while sinks are largely controlled by diffusion and continuous in time, emissions tend to be episodic because they are often mediated by ebullition, which is–on a short time scale–a discontinuous process [35]. In the grassland investigated, the water table was relatively close to the soil surface, and it is well conceivable that the emission bursts occurred from CH_4_ bubbles originating from the saturated zone. A substantial fraction of these bubbles likely travelled relatively quickly to the soil surface via preferential diffusion paths, so that this flux was not buffered. Third, the static chambers trapped localized emissions, resulting in an apparent uptake kinetic due to the re-distribution of CH_4_ in the surrounding soil and possibly also an associated increase in oxidation due to the elevated CH_4_ concentrations. This phenomenon is artificial and would not occur without the chamber. Finally, it is well possible that chamber handling and soil disturbance from human weight triggered the release of bubbles that would otherwise have occurred later (although the static chambers were placed carefully on the pre-installed base rings, and the weight of the person handling the chambers was distributed by a walking grid). Temporary soil compression could also have pushed high-methane air out of parts of the soil pore network where it would have stayed longer otherwise. Indeed, an indication of disturbance-triggered “burst” CH_4_ release could be that the step-increase in concentrations associated with bubble emission often occurred before or just after the first headspace sampling, but rarely after the second sampling. Generally, handling-induced CH_4_ release appears especially critical, since pressure variation can flush near-surface pore volumes (CH_4_ fluxes: [36]; CO_2_ fluxes: [37]), disturbing diffusion gradients that take long to re-equilibrate. Overall, we thus conclude that it probably is not possible to accurately assess the true CH_4_ balance using static chambers in such a system, at least for periods in which net CH_4_ emissions occur. One strategy may be to analyze different processes or different parts of the season independently, using different techniques (e.g. assess continuous fluxes with standard techniques and separately count the occurrence of “burst”-type events).

CH_4_ fluxes exhibited marked seasonal dynamics, with emissions peaking in summer and early fall. While water table depth, soil moisture, and heavy precipitation are likely drivers of these CH_4_ emissions due to their effect on oxygen supply, other factors also may have been at play. High plant activity during peak season could have supplied heterotrophic soil organisms with organic substrate, which would have lowered oxygen partial pressures when consumed–soil CH_4_ oxidation, however, is generally rather limited by CH_4_ concentrations unless O_2_ is nearly depleted, so that seasonal dynamics are unlikely to have been affected by this mechanism. Some organic compounds can also inhibit CH_4_ oxidation directly [18, 19]. Methanogenesis also is strongly temperature-dependent, and it may be that–depending on the zone in which methanogenesis occurred–sufficiently high temperatures were only reached in late summer. Finally, large numbers of Scarabidae larvae are active at the site studied, and incubations of soil cores taken from the site have previously shown that these larvae can release large amounts of CH_4_ [38], a phenomenon that has not received much attention to date for temperate ecosystems.

The nature of methanotrophs capable of growing at atmospheric or sub-atmospheric CH_4_ concentrations remains enigmatic, despite many years of research. Early studies have suggested that methanotrophs predominantly consuming CH_4_ at low or high concentrations differ in nature [39], but it has also been argued that these organisms may be less distinct than previously thought [40]. Indeed, methanotrophs capable to adapt physiologically to environments differing in CH_4_ supply have been found [5], and some possess of isoenzymes differing in kinetic properties [41]. Methanotrophs are alternatingly exposed to low and high CH_4_ concentrations in the studied grassland, depending on whether the atmospheric or soil-internal sources dominate. Our labelling experiments suggest that the methanotrophs actively consuming CH_4_ under these contrasting conditions occupy the same spatial niche. Typically, high CH_4_ concentrations would be supplied from the bottom of the soil column, but our experiments showed that assimilation was nevertheless possible throughout the soil profile, so that this likely did not bias our results. The most abundant CH_4_ oxidizer at our site is a *Methylocystis* strain closely related to a cultured type (LR1) capable of displaying high-affinity kinetics when starved [42]. In this light, it appears well possible that the radiolabel assimilation we observed not only occurred at the same spatial location but that it also was driven by the same type of organisms.

The autoradiographic technique we have developed has not been applied to many sites so far. The patterns we observed, however, were similar to the ones found in the Rothamsted “Park Grass” experiment [43] and in two drought studies [9]. Labelled CH_4_ assimilation concentrated in the periphery of soil features such as aggregates, probably reflecting the ease of diffusive transport to these sites. In October, when soils were wetter, CH_4_ assimilating zones were more concentrated towards the soil surface, and in a smaller part of the pore network (probably macro-pores).

In conclusion, no effects of elevated CO_2_ on net CH_4_ fluxes and the spatial micro-distribution of methanotrophic bacteria were found in the present study. Net CH_4_ fluxes were the result of CH_4_ oxidation and production, with the latter dominating. There are also indications that emissions are mediated by the activity of ground-dwelling arthropods [38] and possibly fungi [44], but the mechanisms involved remain unclear. The range of sources and sinks involved, together with their different dynamic and ecological characteristics, indicate the challenges in estimating a system-level CH_4_ balance and highlight the need to develop a framework in which these fluxes can be constrained; this might include analyzing periods with uptake and emissions separately, constraining these parts of the balance separately

## Supporting Information

S1 Dataset Methane flux data presented in this article. A detailed description of the data is contained in the file.
(ZIP)
